# Case Report: Combined perioperative extracorporeal membrane oxygenation for acute heart failure caused by mitral regurgitation

**DOI:** 10.3389/fcvm.2024.1345654

**Published:** 2024-03-15

**Authors:** Brock Daughtry, John Richardson

**Affiliations:** ^1^ Department of Surgery, Brookwood Baptist Health, Birmingham, AL, United States; ^2^ Department of Cardiothoracic Surgery, Grandview Medical Center, Birmingham, AL, United States

**Keywords:** ECMO, Impella, mitral valve, acute heart failure (AHF), mitral regurgitation

## Abstract

Extracorporeal membrane oxygenation (ECMO) and extracorporeal life support (ECLS) devices are well-established adjunctive treatment measures for patients with heart failure. ECMO can serve as a bridge to transplant in a chronic setting or as a salvage therapy for patients who are unable to be weaned from bypass following cardiac surgery. However, the role of ECMO as a bridge to definitive therapy in a setting of acute heart failure is less established. Similarly, the treatment of patients using combined ECMO and ECLS devices has been, at times, shown to show some benefit; however, these benefits have not been widely studied. In this study, we present the case of a patient who was diagnosed with severe acute onset heart failure secondary to torrential mitral regurgitation following COVID-19 pneumonia. The patient was emergently placed on venoarterial (VA) ECMO with an indwelling centrifugal pump device in the left ventricle. This combination of ECMO and ECLS served as a bridge to open mitral valve replacement 6 days after presentation. Following successful mitral valve replacement, the patient had persistent right ventricular failure, and therefore, a decision was made to incorporate venovenous (VV) ECMO into the VA ECMO circuit. This technique resulted in a VV-VA or VPa-VA configuration, as oxygenated blood was being returned to the pulmonary artery as well as the descending aorta. VA ECMO was discontinued after 4 days of therapy, and the patient was extubated 3 days later. VV ECMO was weaned over the following week, and the patient was decannulated after a total 23 days of ECMO. The patient was then transitioned to inpatient rehabilitation and ultimately discharged home after 18 days. At the 6-month follow-up, the patient was doing well, and objective cardiopulmonary testing revealed normal function. This case is an excellent demonstration of how advanced ECMO and ECLS devices can be used in unique ways through multiple configurations to rescue and optimize patients in the perioperative period.

## Background

Aggressive extracorporeal life support (ECLS) devices have increased in both popularity and availability since their invention in the 1950s. According to the ELSO database, extracorporeal membrane oxygenation (ECMO) devices are now available in over 350 hospitals in the United States (1). The total number of circuits is ever evolving, as are the indications for use. ECMO and ECLS devices have been used in a setting of chronic heart failure and prolonged pulmonary disease, especially as a bridge to transplant, but the use of these treatment measures in a setting of acute heart failure is less established. The ability to stabilize a patient and achieve medical optimization for cardiac surgery after an acute presentation could potentially be a life-saving advancement for those who would have otherwise been unfit for surgery. Additional areas of interest are the variations of different ECMO configurations, ECMO use before and after cardiac surgery, and combining ECMO devices with additional ECLS devices such as balloon pumps or centrifugal pump devices. Combining venoarterial (VA) ECMO circuits with venovenous (VV) ECMO circuits has been shown to be effective in extreme circumstances (2). In this study, we report the case of a 54-year-old man who presented with acute cardiogenic shock due to severe mitral valve regurgitation. He was placed on VA-ECMO with centrifugal pump support until he underwent mitral valve replacement. Following mitral valve replacement, he developed right heart failure and was subsequently transitioned to VV-VA ECMO with the assistance of a dual lumen internal jugular vein catheter. He was ultimately decannulated in a stepwise fashion and discharged home. At the 6-month follow-up, his physical activity returned to baseline and objective testing showed normal cardiopulmonary function.

## Case report

**Figure 1:**
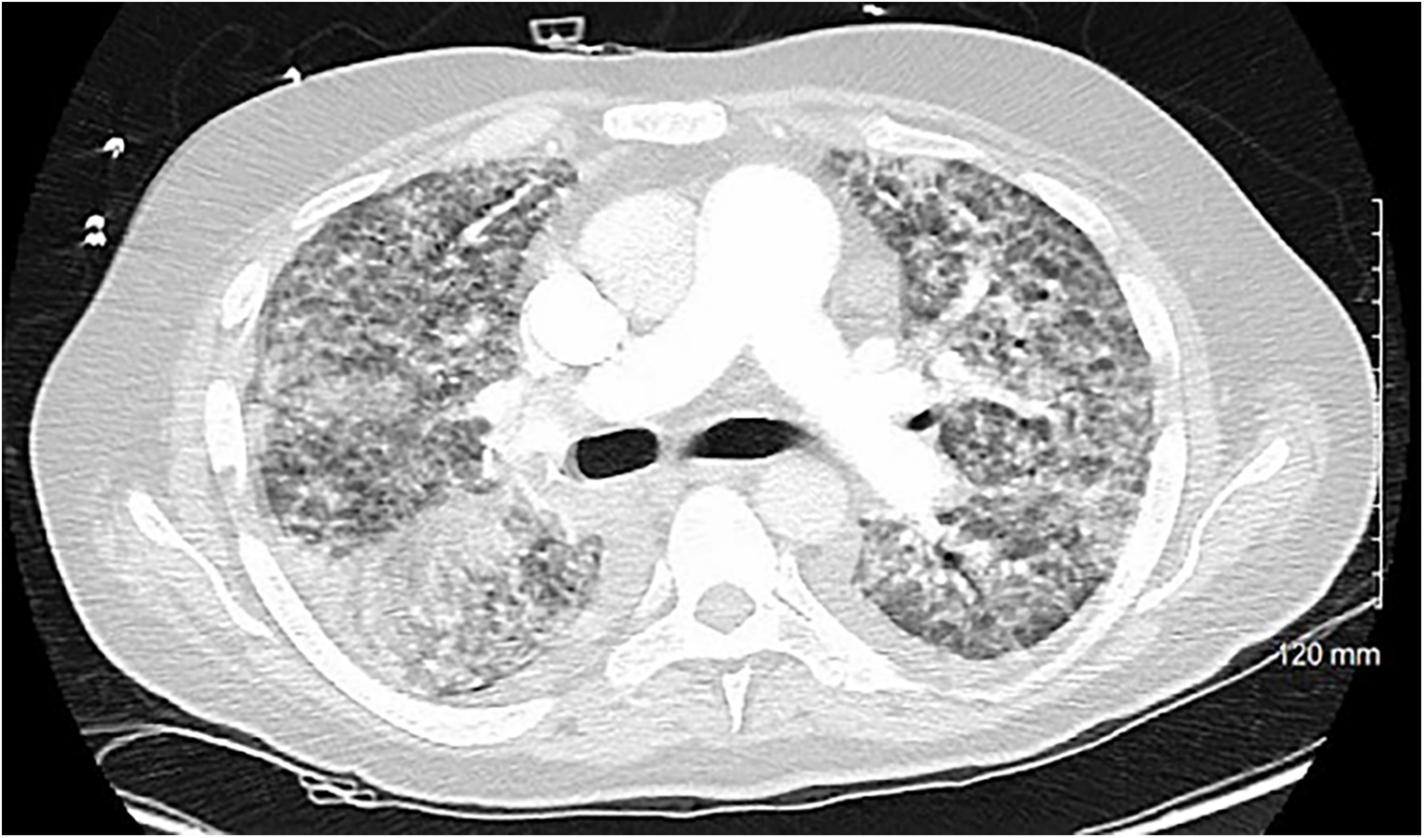
A cross-sectional CT scan performed upon arrival to the emergency department that rules out pulmonary embolism and shows bilateral interstitial consolidations consistent with pulmonary edema.

**Figure 2:**
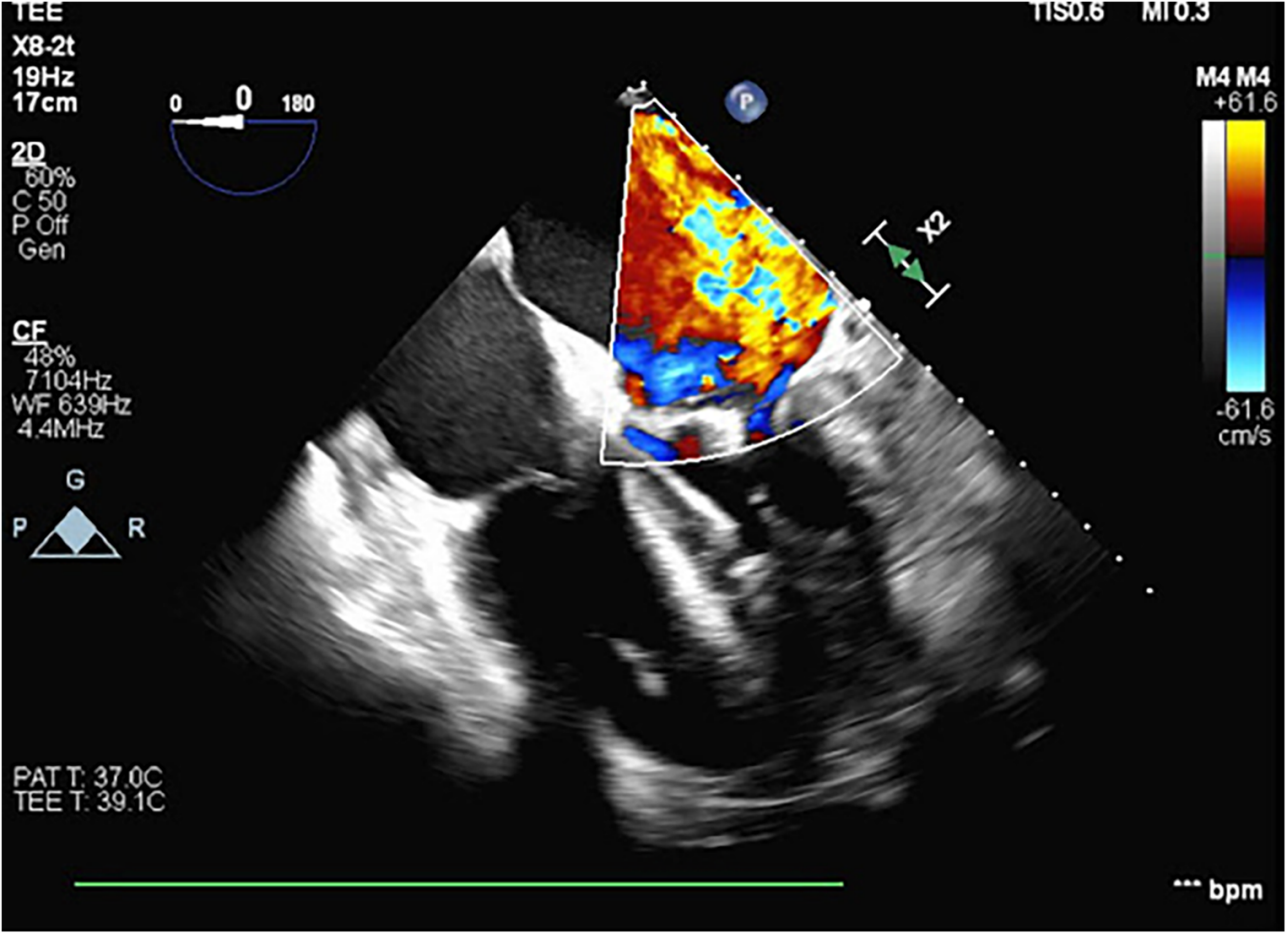
A transesophageal echocardiogram performed during VA ECMO cannulation and Impella placement showing severe mitral regurgitation with the central jet and a thickening of the mitral valve leaflets.

A 54-year-old otherwise healthy man presented to the emergency department with acute cardiogenic shock with respiratory failure (HR 124, SBP 120, RR 38, BNP 15,000) following a 10-day hospitalization 3 months’ earlier with COVID-19 pneumonia. The oxygen saturation rates were 68% on room air and 81% on 5 L of nasal cannula. He was also noted to have a lactic acid level elevated to 4. A computed tomography (CT) scan was performed, which showed diffuse pulmonary edema but ruled out pulmonary embolism (Figure 1). Due to respiratory decline, he was urgently intubated. A transthoracic echocardiogram was obtained, which showed severe mitral valve regurgitation with a flattening of the interventricular septum and a left ventricular ejection fraction of 70%. The mitral regurgitation was described as “severe” and the findings were consistent with endocarditis and papillary muscle rupture (Figure 2).

The patient was taken emergently to the operating room for an initiation of VA ECMO and placement of an Impella® device (Abiomed, Inc., Danvers, MA, USA). Right heart catheterization revealed a pulmonary capillary wedge pressure of 50 mmHg and a right atrial pressure of 20 mmHg. The pulmonary artery oxygen saturation rate was noted to be 36% as measured using a Swan-Ganz catheter. Femoral cannulation ensued with flows of 5.4l and an Impella-assisted cardiac output of 4 LPM. A transesophageal echocardiogram was obtained at the time of ECMO cannulation, demonstrating severe mitral regurgitation with the central jet filling the left atrium. The patient was taken to the cardiac intensive care unit for hemodynamic stabilization and preoperative optimization. While in the ICU[AQ: Please define “ICU” at first occurrence.], metabolic acidosis was corrected, overall volume status improved with diuresis, reaching a negative volume balance of 6 L, and hemodynamics stabilized.

On hospital day 6, the patient was again taken to the operating room for mitral valve replacement. This decision was based on his relative clinical stability at the time, while also appreciating the fact that the mechanical valvular failure that led to his acute decompensation would not resolve without surgical intervention. On the day of surgery, his lactic acid and CO_2_ levels were both within normal limits, his ejection fraction based on transthoracic echo was 60%, and pulmonary artery pressures decreased to 15 mmHg.

The heart was approached via median sternotomy and a peripheral cardiopulmonary bypass was instituted using the existing ECMO cannulas. A cross clamp was placed over the Impella catheter shaft after an echocardiogram confirmed that the drive motor and outflow tract would not be clamped. The Impella device was turned off while on cardiopulmonary bypass. Mitral valve replacement was performed using a 29-mm porcine valve. During the procedure, it was noted that the anterior leaflet of the mitral valve was significantly damaged, the posterior leaflet was necrotic, and there was evidence of infection that could be traced into the left atrium. There was evidence of papillary muscle necrosis but no annular abscess. The final pathology of the specimen showed nodular calcifications measuring 0.6 cm in diameter as well as Gram-positive cocci in chains on the Gram stain. Following replacement of the valve, the patient was placed back on VA ECMO with Impella support. Postoperatively, the patient returned to the ICU on VA ECMO support with RPMs of 6,000 and flows of 4 LPM. The Impella flowed at 0.8 LPM.

Unfortunately, the patient had significant coagulopathy and ongoing mediastinal bleeding requiring transfusion and ultimately needed a return to the operating room for hematoma evacuation and hemorrhage control. Due to this, the patient was unable to be anticoagulated. This led to eventual thrombosis of the Impella device. Flows from the device were negligible, and therefore, it was removed. During removal, the patient was observed with no changes in hemodynamics, and therefore, the device was not replaced.

**Figure 3:**
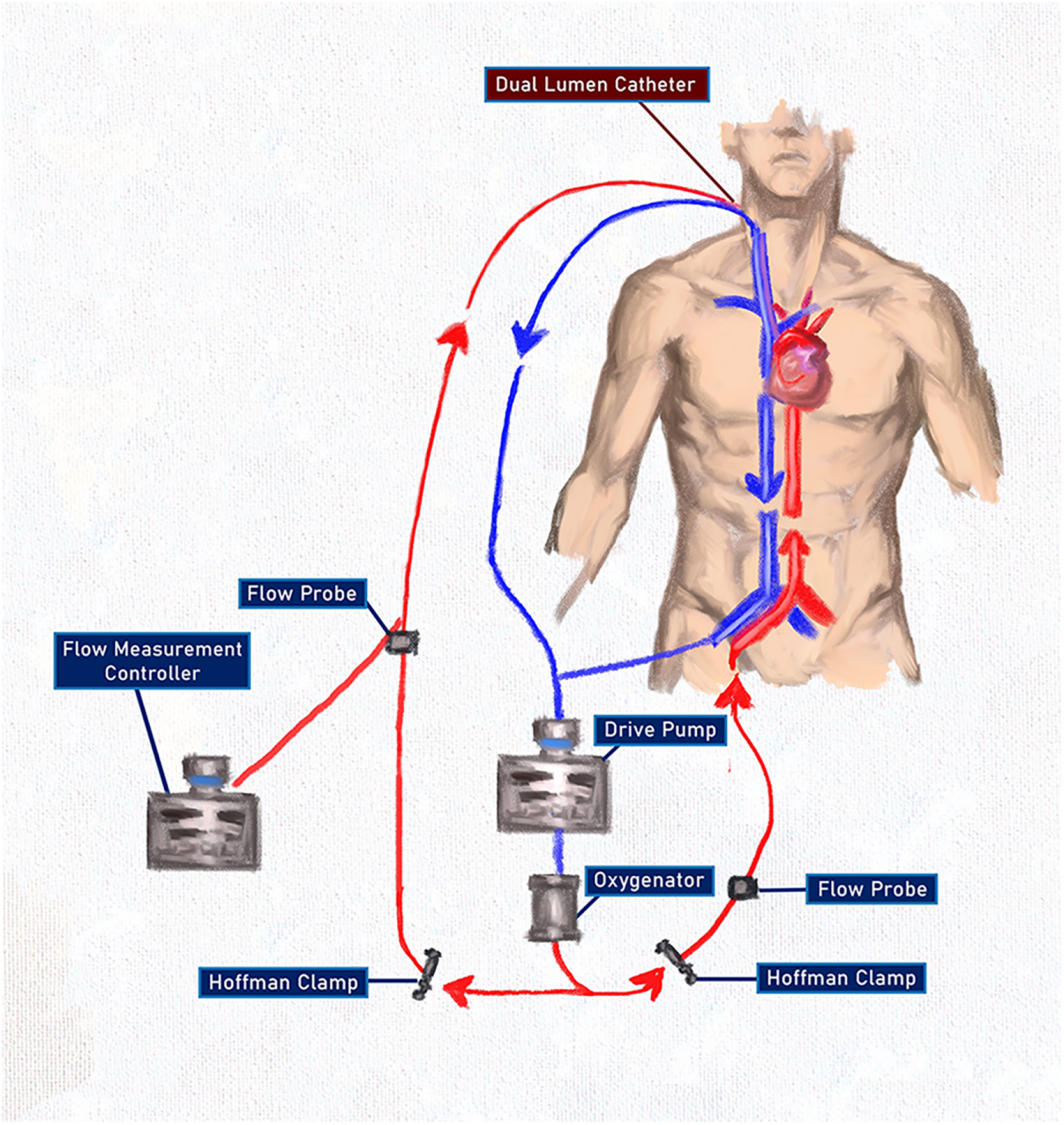
An ECMO configuration after combining VV and VA ECMO using a single pump and oxygenator with oxygenated blood delivered to both the pulmonary and the systemic circulations. The use of the Hoffman clamps allowed for titration of flow through both systems without the complications associated with two competing pumps. The flow measurement controller on the arterial side of the VV circuit was used only as a flow-measuring device and RPMs were not titrated.

On the third postoperative day, a repeat echocardiogram showed an ejection fraction of 20% with a severely reduced right ventricular systolic function. Despite this, and the recent removal of the Impella device, the need for vasopressors saw a decrease. Considering the patient's need for nitric oxide, the evidence of pulmonary edema and fibrosis noted on the initial CT scan, and the concern for prolonged pulmonary support after myocardial recovery, a decision was made to incorporate a VV ECMO circuit into the existing VA ECMO circuit (Figure 3).

A wire was passed down the Swan-Ganz catheter in the right internal jugular vein and guided into the pulmonary artery. Using the Seldinger technique and a series of dilations, a 31Fr Protek Duo® (LivaNova PLC, London, UK) single-site, dual-lumen cannula was inserted under fluoroscopic guidance. This provided venous drainage from the right atrium and oxygenated arterial return into the pulmonary artery, bypassing and unloading the right ventricle. The venous drainage was connected in a Y fashion to the venous line of the VA ECMO circuit, allowing for venous drainage from the right atrium and the right femoral vein. Arterial return was now directed to the pulmonary artery via the VV circuit at the aorta via the VA circuit. Flows through both circuits were set to 3 L with stable hemodynamics. After the patient returned to the ICU, the VA ECMO circuit was titrated to 6,500 RPM, creating a flow of 4.8 LPM. Using a Hoffman clamp for outflow restriction, the VV ECMO circuit was titrated to flows of 2.9 LPM with a sweep rate of 2.5.

Over the following days, flows for the VA circuit were weaned by titrating both the pump speed and the Hoffman clamp occlusion. Flows through the VA limb of the ECMO circuit were decreased to less than 2 LPM of flow for several days, and the patient was able to be weaned from epinephrine. Ventilator requirements were now significantly reduced to very low tidal volumes and pressures. Four days after the placement of the Protek Duo cannula, the patient was decannulated from VA ECMO. An echocardiogram at that time demonstrated a left ventricular ejection fraction of 45% with minimal milrinone support.

**Figure 4:**
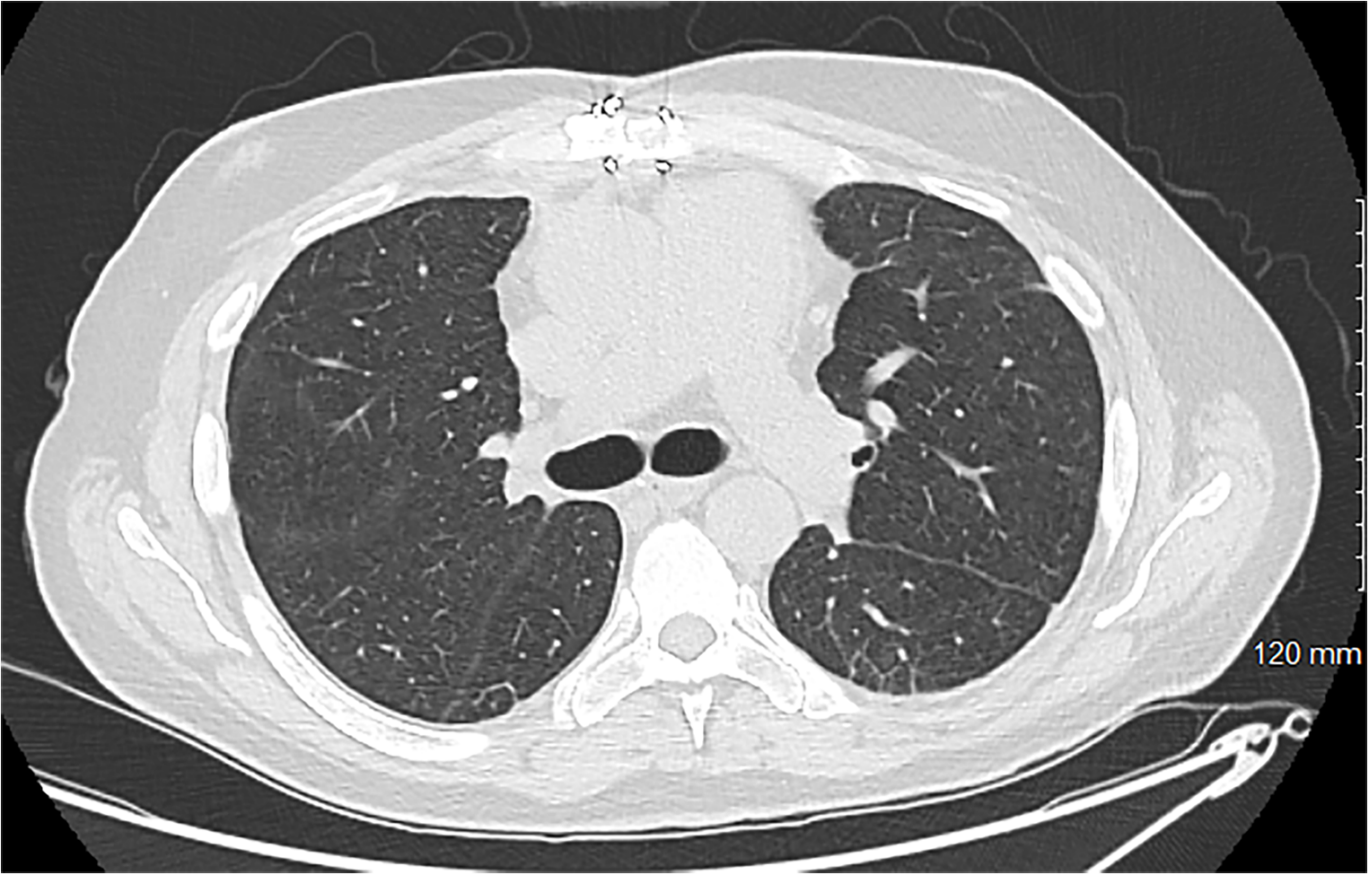
A cross-sectional CT scan obtained for a 3-month follow-up showing minimal basilar scarring with complete resolution of the previously noted edema. More inferior images show a well-placed mitral prosthesis and a well-approximated sternum.

Three days after VA decannulation, the patient was extubated and began working with physical therapy and ambulating while on VV ECMO. Flows through the Protek system at that time ranged between 2 and 3.5 LPM of flow. Eventually, 14 days after the Protek Duo cannula was placed, the patient was decannulated. After another week of optimization and continued critical care, the patient was discharged to inpatient rehab. The total hospitalization time was 31 days with a total ECMO time of 23 days. The patient spent 18 days in rehab and was then discharged home. The patient was doing well and reported only slight dyspnea with exercise. Follow-up testing showed adequate pulmonary recovery both radiographically and on pulmonary function tests (Figure 4).

## Discussion

This case first highlights the preoperative use of ECLS devices to optimize and bridge to eventual cardiac surgery in a setting of acute cardiogenic shock. The strategy of bridging is common and well established for patients with chronic heart failure needing a transplant, but its use in the acute setting is less well established and is mostly shown in case reports and small case series. Ekanem et al. describe a three-case series of VA-ECMO and Impella support for cardiogenic shock following postinfarct papillary muscle rupture (3). They successfully bridged two of three patients to eventual surgery, both of whom survived their hospitalization period. ECMO alone has also been proved to serve as a successful bridge to definitive operation (4–6). In all of these cases, the patients underwent successful surgery and were discharged from the hospital. Coyan et al. provide an excellent review of VA-ECMO and Impella in their 2022 review of 25 studies compiling 83 patients for whom ECLS strategies were employed following a postinfarction ventricular septal rupture (7). In our patient in this study, the expedient initiation of ECLS devices proved to be both a life-saving measure and a successful bridge to eventual cardiac surgery. During that time, he was on maximal medical support including VA ECMO, Impella therapy, mechanical ventilation with moderate ventilator requirements and inhaled nitric oxide, as well as multiple vasopressors and inotropes for hemodynamic support. The time between initiation of the initial configuration of VA ECMO and Impella was used to optimize the patient for mitral valve replacement to a point of medical stability. It was felt that there was a narrow window for intervention during this improved state before the effects of the significant mitral regurgitation could not be overcome despite maximal support.

Another aspect of ECLS underscored by this case is the hybrid use of ECMO configurations for additional cardiopulmonary support. Brasseur et al. best described the ECMO configuration used in this case as VPa-VA ECMO (8), highlighting that not only was venous blood drained from the right atrium and the inferior vena cava as in VVA, but that oxygenated blood was being returned to the pulmonary circulation via the Protek Duo cannula and to the systemic circulation via the VA system. Camboni described similar blood flow using a surgical approach requiring sternotomy (9); however, the construction described here achieved the same blood flow through a percutaneous approach. Reconfiguring and combining ECLS systems has proved to be successful in several scenarios previously. Matsuyoshi et al. described transitioning from VA to VAV to VV ECMO following cardiac arrest due to massive pulmonary embolism with eventual decannulation and discharge (2). Haldenwang and colleagues presented eight patients treated with combined cardiac and pulmonary failure who underwent initial VA ECMO and were then transitioned to VVA and ultimately to VV ECMO prior to being weaned (10). They were able to successfully wean 75% of patients and achieve a 30-day survival rate of 50%. The notable difference in the case presented here when compared with the previously mentioned studies is that oxygenated blood was returned to both the systemic system and the pulmonary systems, while allowing myocardial rest and recovery for both the right and the left heart. This configuration provided both right ventricular support and assistance with oxygenation, while also being able to wean, and eventually remove, the left ventricular support. Given our unique situation of pulmonary insult from both recent COVID-19 pneumonia and significant pulmonary edema from the defective mitral valve, the continued pulmonary support offered by the VV ECMO circuit proved vital and allowed for lung protective ventilation and earlier extubation, initiation of ambulation, and likely earlier discharge.

This case highlights several complex perioperative factors that influence patient outcomes when dealing with cardiogenic shock, as well as salvage measures that can be employed. Cardiogenic shock due to acute valvular emergencies is both a medical and a surgical emergency, with an operative mortality rate reaching as high as 22% (11). Sperry et al. describe the cardiothoracic surgeon's role specifically, stating, “surgeons play a critical role in the management of cardiogenic shock due to their contributions to both the short-term and long-term decision making.” They reference the cardiac surgeon's expertise in deranged cardiac physiology, familiarity with a wide range of devices, and ability to offer definitive therapies as reasons why surgeons should be involved immediately when cardiogenic shock is diagnosed (12). In the case previously described, early involvement of the cardiac surgical team led to a prompt initiation of ECLS and subsequent management and reconfigurations both pre- and postoperatively.

## Conclusion

The ability to rescue acutely ill patients at the time of presentation allows valuable time for patient optimization and transition to surgery. Likewise, postoperative use of ECLS devices in different configurations can allow time for cardiac and pulmonary recovery and potentially lead to better outcomes for all patients. In our patient, VA ECMO combined with an Impella device was used to optimize the patient for cardiac surgery. Postoperatively, right ventricular recovery was impeded by the significant pulmonary edema, and therefore, a Protek Duo cannula was placed and joined with the VA ECMO circuit to facilitate right ventricular off-loading, while providing oxygenated blood to both the pulmonary and the systemic circulations. As the left heart continued to improve, VA ECMO was removed, and the patient was extubated, allowed to ambulate, and eventually decannulated entirely. This case proves the utility of early ECMO initiation and the potential benefit of different configurations of ECLS devices to improve patient outcomes.

## Data Availability

The raw data supporting the conclusions of this article will be made available by the authors without undue reservation.
